# Central intracrine DHEA synthesis in ageing-related neuroinflammation and neurodegeneration: therapeutic potential?

**DOI:** 10.1186/s12974-018-1324-0

**Published:** 2018-10-16

**Authors:** Y S L Powrie, C Smith

**Affiliations:** Department of Physiological Sciences, Stellenbosch University, Private Bag X1, Matieland, Stellenbosch, 7602 South Africa

**Keywords:** Steroidogenesis, Extragonadal, Accelerated ageing, Alzheimer’s, Immunosenescence, Sulphotransferase, Translocator protein, Species-specific, Neuroprotection, Antioxidant

## Abstract

It is a well-known fact that DHEA declines on ageing and that it is linked to ageing-related neurodegeneration, which is characterised by gradual cognitive decline. Although DHEA is also associated with inflammation in the periphery, the link between DHEA and neuroinflammation in this context is less clear. This review drew from different bodies of literature to provide a more comprehensive picture of peripheral vs central endocrine shifts with advanced age—specifically in terms of DHEA. From this, we have formulated the hypothesis that DHEA decline is also linked to neuroinflammation and that increased localised availability of DHEA may have both therapeutic and preventative benefit to limit neurodegeneration. We provide a comprehensive discussion of literature on the potential for extragonadal DHEA synthesis by neuroglial cells and reflect on the feasibility of therapeutic manipulation of localised, central DHEA synthesis.

## Background

From the recent literature, it is evident that the processes of neuroinflammation and neurodegeneration are inextricably linked. Given the sequestered nature of the brain, which complicates research sample collection for obvious reasons, many investigators seem to extrapolate data generated from peripheral samples in attempts to explain central events. However, as also illustrated in the pages to follow, there is often a disconnection between adaptation in the periphery versus those occurring centrally. In our opinion, there are multiple reasons for this. Firstly, the neuroimmune system is structurally distinct from the peripheral system in that most immune functions are mediated by cells specific to the nervous system, such as microglia and astrocytes [1]. Incidentally, although recent commentaries and research letters pertaining to the identification of a lymphatic system in the dural spaces are suggesting that the brain may be subject to surveillance by immune cells circulating from the periphery [2], not enough data exist with which to evaluate the relative importance of these immune cells relative to those residents in the brain. Secondly, the brain has a preference for glucose as a substrate, as opposed to most peripheral organs, such as the heart, which mainly derive energy through fatty acid β-oxidation, which may affect the outcome of adaptive—or maladaptive—metabolic responses differently in the brain to peripheral compartments. Lastly, although the brain itself is subject to glucocorticoid-mediated metabolic modulation, as in the peripheral compartment, it is also directly affected by—and thus adapts as result of—psychological input.

From this, it is clear that different factors come into play centrally vs. peripherally in terms of adaptation to stimuli. This questions the validity of treating neuroinflammation—especially in the context of chronic disease aetiology—using the same strategies by which peripheral chronic low-grade inflammation is addressed. It further highlights the need for specific, central investigations for the purpose of answering questions pertaining to central physiological adaptation or maladaptation.

We have identified the hormone (also aptly referred to as a neurosteroid) dehydroepiandrosterone (DHEA) as a relatively under-researched hormone in the context of neurophysiology, despite its very clear association with a plethora of clinical disease states. DHEA, which is predominantly synthesised in the human adrenal cortex, also exists as a sulphated ester known as DHEAS, which is formed when DHEA is processed by the enzyme steroid sulphotransferase (SULT) [3, 4]. Of note, the majority of DHEA in circulation exists as DHEAS due to it having a stronger binding affinity for its carrier protein, albumin [5]. DHEA is the precursor hormone to both androgenic and estrogenic hormones and, as such, is also synthesised to a smaller extent in human gonads. In addition, and most relevant to the context of the current topic, it is claimed to be synthesised de novo in the human brain as well. In fact, DHEA concentrations in the human brain have been shown to be higher than that in circulation, while DHEAS concentrations are lower [4, 6], which not only supports the theory of local synthesis, but also testifies to the importance of this hormone centrally.

In the context of human health, DHEA has historically been deemed to have limited importance, until low circulating levels of DHEAS were associated with chronic and age-related diseases such as diabetes, hypertension and arthritis. [7–11]. In addition, it has since drawn much media attention as being an “anti-ageing” supplement after circulating levels were also reported to dramatically and progressively decline with age [9, 12]. Most relevant to the current context, abnormal changes in DHEA levels in serum and cerebral spinal fluid (CSF) have also been reported in the pathophysiology of neurodegenerative diseases such as Alzheimer’s disease (AD), schizophrenia and multiple sclerosis (MS), as well as neurocognitive pathologies such as post-traumatic stress disorder (PTSD) (a summary of these reports is provided in Table 1). However, much remains unknown about DHEA’s mode of action in the human body or the reason for its age-related decline. Therefore, and given the commonly accepted phenomenon of accelerated ageing in many chronic disease states [13], understanding the process of ageing and the physiological role of DHEA in this process, may be of benefit to the development of novel strategies in the treatment of these enigmatic pathologies.

**Table 1:** Summary of reports linking DHEA to neurodegenerative diseases and neurocognitive disorders

Disease	Findings	References
Alzheimer’s disease	Significant decrease in serum DHEA and DHEAS levels when compared to aged-matched control patients	[165–171]
Schizophrenia	Data on both sides of the spectrum associate both abnormally elevated and declining levels of DHEA/DHEAS with the disease. This opposing data could be related to the heterogeneity of the disease itself, as well as other comorbid aetiological factors, which adds complexity to interpretation of this data.	[172–178]
Multiple Sclerosis	Significantly higher CSF DHEA concentrations in relapsed patients relative to control patients with stable neurological disease	[179]
Post-traumatic stress disorder (PTSD)	Increased plasma DHEA and DHEAS levels when compared to unaffected control patients	[180–186]

With this review, we provide a comprehensive summary of what is known about the role of DHEA in the context of neurophysiology and ageing. This will be followed by a discussion of specific topical issues, such as the effect of sex and species, as well akowski the sites for DHEA production. More specifically, relevant literature providing arguments for and against central, intracrine DHEA production is discussed in order to make an interpretation on the likelihood of central DHEA biosynthesis taking place, as well as on the feasibility of modulating central DHEA levels for therapeutic effect in the context of ageing-related neuroinflammation and ageing-related degeneration.

## Process of ageing

Ageing is an inevitable, natural biological process characterised by a progressive loss of physiological integrity which results in impaired function and increased probability of death. It is an extremely complex process which is affected by a number of lifestyle and genetic factors that may accelerate, ameliorate or even slow down the progression of ageing itself [13]. The process of ageing is commonly considered the main risk factor in the development of neurodegenerative diseases such as Alzheimer’s disease and Parkinson’s disease (PD) [14, 15]. Currently, the global population is living longer as a result of the advances in medicine and, consequently, such diseases are becoming more prevalent.

Ageing itself is associated with cumulative oxidative stress, chronic low-grade inflammation linked to age-related dysfunction of the immune system (termed immunosenescence) and most notably, a decline in endocrine function (termed endocrinosenescence) [13, 16]. The immediate sections to follow will focus primarily on immunosenescence and endocrinosenescence—highlighting their intricately linked aetiologies in the process of ageing itself, as well as in age-related neurodegenerative pathologies.

### Immune system maladaptation

Inflammation is an integral part of the innate immune system: it not only acts as the first line of defence against pathogenic attack and injury, but also serves to execute final humoral immune effects (e.g. antibody-mediated pathogen destruction). It is thus a normal physiological process that is essential for the maintenance of homeostasis. An equally normal consequence of the inflammation-facilitated repair of tissues after any insult is the transient disruption of the local cellular homeostasis of non-infected or uninjured cells. This injury-repair cycle is efficient during youthful years, but is affected by ageing, resulting in a relatively impaired ability of the body to regenerate damaged tissues [13]. Systemically, immunosenescent changes in the innate immune system are characterised by a loss of phagocytic capacity, reduced efficiency in leukocyte (neutrophil and macrophage) chemotaxis, reduced intracellular, but increased extracellular levels of free radicals and an increase in production of “early” or acutely responsive pro-inflammatory cytokines, such as interleukin (IL)-1β, IL-6 and tumour necrosis factor (TNF)-α [17–20], all of which contribute to a more pro-inflammatory phenotype and which increase the burden of tissue damage-recovery cycles. The loss in efficiency of this process, coupled with an increased exposure to antigenic load and modern lifestyle associated chronic stressors, results in the reduced ability of the body to maintain normal immune function. This manifests as an increased risk for malignancy, auto-immune responses, an impaired ability to mount proper immune responses to stimuli and prolonged inflammatory activity during acute infections [21–23].

#### Neuroinflammatory changes

As in the periphery, ageing of the brain is associated with increasing inflammation and local oxidative stress—also resulting in repeated tissue damage-recovery cycles—which predisposes older individuals to developing neurodegenerative pathologies. Age-related neuroinflammation partly manifests as an increased reactivity of microglia, the resident innate immune cells. In a healthy brain, microglia are activated upon stimulation, but do not necessarily release these pro-inflammatory mediators. In the aged brain however, microglia appear to not only release pro-inflammatory mediators, but to do so in an exaggerated and prolonged manner [24]. This appears to be linked to the fact that microglia undergo significant immunophenotypic and functional changes with ageing: the activation status of microglia changes from a quiescent to a “primed” state. This manifests as an increased expression of the glial activation markers major histocompatibility complex II (MHC II) and complement receptor 3 (CD11b), which sensitises microglia to the exaggerated pro-inflammatory responses seen upon stimulation.

Animal studies have also suggested that chronic and significant elevation of levels of pro-inflammatory cytokines such as IL-1β may be linked to impaired memory formation and long term potentiation (LTP) [24–27]. Systemically, IL-1β is released by monocytes upon recognition of a pathogen or site of injury, as well as by any cell sustaining damage [28]. IL-1β enhances B and T cell lymphocyte proliferation and stimulates IL-6 and TNF-α production in many other immune cells. The systemic elevation of circulating pro-inflammatory cytokines, particularly IL-1β, exerts a significant effect on the microglial activation as well. However, in this context, the age-related maladaptation seems to occur at different magnitudes in the brain relative to the circulation. For example, studies in mice have shown that stimulation of the peripheral innate immune system with an intraperitoneal injection of either lipopolysaccharide (LPS) or live *E.coli* bacteria resulted in a more prolonged and exaggerated elevation of IL-1β and IL-6 levels in aged brains relative to those of younger mice [29, 30]. Interestingly, the exaggerated and prolonged inflammatory response was restricted to the brain and not paralleled with a similar peripheral response. In addition, it was reported that the hippocampus was more affected when compared to other brain regions, such as the hypothalamus, parietal cortex or prefrontal cortex [30]. The hippocampus plays a critical role in memory formation, and consequently, it is implicated in many neurodegenerative pathologies, such as Alzheimer’s disease. These data give credibility to the more recent suggestions that a severe inflammatory episode may trigger development of Alzheimer’s disease [31, 32]. It also identifies the hippocampus specifically as area of interest for preventative intervention.

However, before one can move on to interventions, it is necessary to more fully understand the cause(s) of this exaggerated neuroinflammatory outcome upon ageing. A complexity is that the exact cause of the overactive response in microglia from aged individuals has in fact not been elucidated convincingly. We believe that the answer might lie in the progressive and chronic elevation of glucocorticoid hormones that is also associated with ageing.

### Endocrine dysfunction

#### The ageing hypothalamic–pituitary–adrenal axis (HPA)

Cortisol released into circulation can readily cross the blood–brain barrier (BBB) and therefore can exert its effects both locally (in the brain) and systemically (in the periphery), by binding to either of its two intracellular receptors: mineralocorticoid receptor (MR) or glucocorticoid receptor (GR) [24]. A study in rats showed that MRs have a significantly higher affinity for corticosterone (the most abundant endogenous glucocorticoid in rodents) than GRs; the latter is consequently only bound by corticosterone upon complete saturation of MRs during periods of highly elevated serum corticosterone [33]. This phenomenon of preferential MR binding by the endogenous glucocorticoid cortisol (the most abundant one in humans) is also well-documented in humans [34]. Binding of cortisol to its receptors causes it to translocate to the nucleus and decrease gene transcription of pro-inflammatory cytokines—effectively the body’s most important anti-inflammatory mechanism. The brain expresses high levels of both MRs and GRs, with highest MR expression mainly in the hippocampus, which has the highest of MR to GR ratio in the brain overall [35].

To return to the context of this review, rat hippocampal long-term potentiation (LTP), a correlation of synaptic plasticity and strength (and thus capacity for memory formation), has been shown to be suppressed by GR activation [36], which highlights this pathway as role player in the pathology related to neurodegeneration, such as Alzheimer’s disease. The hippocampus is a brain region that is frequently highlighted in neurodegeneration research. Of note, and as mentioned earlier, it is affected more by peripheral inflammation than the rest of the brain. Therefore, should the hippocampus be specifically targeted in the treatment of inflammation-related neurodegeneration? In line with this question, how exactly do glucocorticoids link ageing, inflammation and neurodegeneration? We put forward the following hypothesis: as one ages, the repeated injury-repair cycles require repeated glucocorticoid mediated anti-inflammatory responses. Initially, the hippocampus is sensitive to glucocorticoids due to a high expression of GR and MR receptors. However, after numerous glucocorticoid responses, the hippocampus downregulates receptor expression—something which is indeed seen in acute and chronic stress [37–40]. In fact, more than 30 years ago, Sapolsky and colleagues reported aged rats to have elevated basal corticosterone levels and an impaired capacity for levels to return to basal after an acute stress response [41]. This landmark paper proved to be the basis of the glucocorticoid hypothesis, which postulated that increasing age correlated with gradual loss of negative feedback control resulting in the gross accumulation of cortisol/corticosterone not only systemically, but also in the brain [42]. The loss in negative feedback may be attributed to a decrease in MR and GR expression in the brain. For example, in cases and animal models of neuropsychiatric diseases in which patients present with hypercortisolemia, such as major depressive disorder and PTSD, MR expression has been found to be downregulated specifically in the hippocampus [39, 43]. A final possibility that we propose is that MR and GR activation may be insensitive to glucocorticoid binding in aged human hippocampi. For example, it has been demonstrated in human peripheral blood mononuclear cells that in cells expressing high levels of GRs, GR affinity for dexamethasone (a synthetic corticosteroid) is significantly reduced when compared to individuals with peripheral blood mononuclear cells (PBMCs) with lower expression levels [44].

Given the age-related exaggerated and prolonged inflammatory responses discussed above, an exaggerated anti-inflammatory counter in the form of increased glucocorticoid levels is probably to be expected. However, the problem with the pro- and anti-inflammatory system crosstalk lies in that this exaggerated response, which is well-recorded in the ageing literature, is rendered insufficient to counter and resolve the inflammatory response by the endocrine maladaptation discussed here.

#### The effect of age on the sympatho–adrenal–medullary pathway (SAM pathway)

In parallel to its role in the HPA axis, the hypothalamus also plays a critical role in the release of catecholamines, such as epinephrine and norepinephrine from the adrenal medulla, facilitated by sympathetic innervation of the medulla [45]. Importantly, the context of this review is the fact that catecholamines induce both the systemic and localised upregulation of IL-6 synthesis, which can prompt adrenocortical release of glucocorticoids [46–50]. In addition, epinephrine and norepinephrine act synergistically with cortisol by upregulating glucose metabolism and increasing cardiac output during acute periods of stress, i.e. the “fight or flight” response.

Similar to the HPA axis, ageing also affects the SAM pathway, mainly due to changes in the sympathetic nervous system (SNS). Evidently, it appears that overall tonic SNS activity increases with age [51]. Circulating norepinephrine levels appear to increase whereas epinephrine levels decline with age [51–55]. Whether these observations are due to impaired clearance or an increased output by the adrenal medulla is still unknown. However, it is also probable that the increasing circulating concentrations of norepinephrine may in part be due to a spill over from synaptic junctions at effector sites—which has already been reported in at least one study [51]. Although no consensus has been reached with regard to why these changes may occur with age, it is very clear that they do occur.

When compared to the vast knowledge gathered on the effects of age on cortisol and the HPA axis, relatively little is known about the SAM pathway and even less so about its effects on the inflammatory process. However, hormones in the SAM pathway have demonstrated immune modulating behaviour. For example, epinephrine has been shown to potently inhibit LPS-stimulated human monocytes from synthesising pro-inflammatory cytokines, such as TNF-α and IL-12, as well as to stimulate the synthesis of the anti-inflammatory cytokine, IL-10 [56]. On the other hand, in the brain, norepinephrine may have a pro-inflammatory effect in the hypothalamus. For example, norepinephrine was shown to play a critical role in foot shock stress-induced hippocampal and splenic production of IL-1β [48]. Pre-treatment with the selective beta-adrenergic receptor antagonist, propranolol, abrogated the IL-1β response. Furthermore, when animals were treated with the microglial inhibitor, minocycline, upregulation of IL-1β was completely inhibited in the hypothalamus, but not the spleen [48]. These results are extremely relevant in the context of ageing, since it is known that microglia play a critical role in age-related neuroinflammation and the fact that noradrenaline levels increase with age.

#### The effect of ageing on sex hormones

The HPA and SAM pathways are not the only endocrine systems affected by ageing. It is commonly known that the most evident effects of ageing occur through a decline in sex hormone production. With regard to a decline in androgen production and the synthesis pathways involved, the female menopause is probably best characterised—in particular, the complete cessation of oestrogen production by the ovaries [57]. Thus, after menopause, DHEA becomes the major androgenic hormone in the female circulation. Interestingly, a recent review suggested that in aged individuals, DHEA—although not able to facilitate ovarian oestrogen production—may be able to serve as precursor for tissue-specific oestrogen synthesis through intracrine mechanisms [57]. According to this theory, oestrogen would be synthesised in low concentrations at tissue level (in any tissue where all the required steroidogenic enzymes are present), where it would act in para- or autocrine fashion, with little or no clearance into circulation. Similarly, in males, extragonadal male androgen production has also been demonstrated in peripheral tissue [58]. With this in mind, a decline in adrenal DHEA production with advancing age would therefore not only have implications for reproduction, but also potentially for other detrimental effects related to a decrease in extragonadal oestrogen or testosterone production at peripheral tissue level.

The decline of DHEA with ageing has been well-established. Peak DHEA levels have been reported to occur slightly earlier in life for females when compared to males (15–19 vs. 20–24 years) [12], after which it steadily declines, reaching levels of ≈20% of peak levels after age 70 [59]. Epidemiological studies have correlated DHEA levels to longevity in both humans and non-human models [60, 61], while the decline of DHEA has been linked to a number of ageing-associated characteristics, including immunosenescence, cognitive decline, osteopenia and sarcopenia [59].

From what has already been discussed, it is clear that ageing is linked to several adaptations/maladaptations in the endocrine system. What will become equally clear from the literature to follow is the relative absence of directly relevant data on DHEA, its involvement in these processes and thus the effects its decline might have. From studies conducted in contexts other than ageing, it will become clear that DHEA has a significant role to play. We discuss these potential roles below.

## DHEA—the magic bullet?

### Pre-clinical evidence suggesting neuroprotective effects of DHEA

Since the turn of the century, several papers suggesting beneficial effects after DHEA supplementation, in the context of neuroprotection, have been published—we have summarised findings illustrating the variety of models used in Table 2. Reported benefits include modulation of neurogenesis, neuronal function, metabolism and longevity. However, a significant limitation to available information is that the majority of these effects have only been illustrated in in vitro and animal studies.

**Table 2:** Evidence for beneficial effects of administered DHEA/DHEAS or metabolites reported in pre-clinical studies

Demonstrated beneficial effects	Reference
General neuroprotective effects demonstrated both in vitro and in vivo in:	
E18 Sprague–Dawley rat hippocampal cell culture model of *N*-methyl-*D*-aspartate (NMDA) neurotoxicity	[187]
HT-22 mouse hippocampal cell line model of glutamate and amyloid-β neurotoxicity	[188]
E18 Sprague–Dawley rat cerebral cortical cell culture anoxia model	[189]
Mouse hippocampal neurodegeneration model	[190]
Rats with induced forebrain ischemia model	[191]
Rat hippocampal slice culture ischemia model	[192]
Primary rat cerebellar granule cell anoxic and glucose deprivation model	[193]
Mouse spinal cord ischemic injury model	[194]
Rat inflammatory neurodegeneration model	[195]
P19 neuronal NMDA-induced excitotoxicity cell line model	[87]
Aged rat brain model	[196]
Rat Corpus striatum (CS) and the nucleus accumbens (NAc) model of assessing effects of DHEA on monoamine oxidase (MOA) activity	[197]
Human SH-SY5Y neuroblastoma cell line model assessing the effects of neurosteroids on mitochondrial bioenergetics	[198]
Rat brain 3-nitropropionic acid (3-NP) induced neurotoxicity model	[199]
Transient brain ischemic mouse model	[200]
Primary male and female mouse cultured hippocampal neurons, as well as human SH-SY5Y neuroblastoma cell line glucose deprivation model	[201]
Anti-apoptotic effects demonstrated in vitro in:	
Undifferentiated P19 neuronal cell line model of NMDA-induced apoptosis	[202]
PC12 rat pheochromocytoma cell line model of serum deprivation-induced apoptosis in adrenal medulla cells	[203]
Primary rat embryonic cultured neural precursor cell model investigating the effect of DHEA and DHEAS on Akt phosphorylation	[204]
Primary rat cerebellar granule cell model of hypoxia and glucose deprivation	[205]
Anti-glucocorticoid effects demonstrated both in vitro and in vivo in:	
Primary rat embryonic hippocampal neurons exposed to neurotoxic doses of corticosterone	[206]
HT-22mouse hippocampal cell line model of glutamate and amyloid-β-induced neurotoxicity	[188]
Mitigating effects on corticosterone-induced suppression of neurogenesis and survival of new neurons in dentate gyri (hippocampi) of Lister Hooded Rats	[207]
Inhibitory effects of DHEA on glucocorticoid amplification in 3 T3-L1 adipocyte cell line and C57BL/6J mouse white adipose and liver tissue	[208]
Inhibition of 11β-hydroxysteroid dehydrogenase 1 (11β-HSD1) mediated conversion of cortisol by DHEA metabolites in human skin samples	[209]
Suppression of 11β-HSD1 mRNA in HEK-293 rat cortical collecting duct cell line, as well as kidneys of C57BL/6J mice and Sprague–Dawley rats	[210]

It is clear that DHEA is neuroprotective within these various disease model contexts, but how is DHEA relevant to inflammation—and in particular to neuroinflammation-related neurodegeneration?

### Direct and indirect evidence for neuroprotective anti-inflammatory effects of DHEA

To the best of the authors’ knowledge, very little evidence for direct anti-inflammatory action of DHEA or DHEAS in the brain or central nervous system (CNS) has been elucidated per se. We were however able to find at least three studies that have demonstrated the anti-inflammatory effects of DHEA in vitro in models relevant to the brain or CNS. The first demonstrated that DHEA inhibits the synthesis of TNF-α and IL-6 by cultured foetal rat astroglia in response to exposure to *Mycoplasma fermetans* [62]. The second and third studies, which are also the most recent, were performed both in vitro and in vivo [63, 64]. In the in vitro study, DHEA treatment of cultured mouse microglia exposed to LPS showed a significant reduction in TNF-α, IL-6, IL-12 and monocyte chemoattractant protein 1 (MCP-1) mRNA expression when compared to LPS-exposed microglia receiving no treatment [64]. The in vivo study was an experimental model of autoimmune encephalomyelitis (EAE) in mice, which showed a significant reduction in IL-1β and interferon gamma (IFN-γ) mRNA expression in spinal cord tissue in animals treated with DHEAS when compared to untreated EAE-affected animals [63].

Numerous other studies have illustrated the anti-inflammatory action of DHEA and DHEAS in systems and disease models other than those related to the brain or CNS (Table 3). Although this does not necessarily prove that these same effects may be seen in the brain under the same or similar circumstances, the possibility cannot be discounted.

**Table 3:** Studies which have demonstrated anti-inflammatory effects of DHEA or DHEAS outside of the CNS

Disease/model	Reference
Reduced regulation of IL-6 production in aged mice	[211]
Reversed effects and decreased production of IL-4 and IL-6 in antigen induced immunosuppression in mice	[212]
Reduced production of IL-6 in splenocytes of retrovirus infected mice as well as aged-induced immunocompromised mice	[213]
Decreased production of IL-4 and increased production of IL-2 by concanavalin-A-stimulated PBMCs from patients with atopic dermatitis	[214]
Reduction of TNF-α serum concentrations in obese Zucker rat model	[215]
Inhibition of IL-6 production in isolated primary human peripheral blood mononuclear cells (PBMCs)	[10]
Reduced production of IL-1, IL-6 and TNF-α in LPS-stimulated murine macrophage cell line	[216]
Inhibition of TNF-α-induced nuclear factor kappa-light-chain-enhancer of activated B cells (NF-κB)-mediated gene transcription in HuH7 human hepatocyte cell line	[217]
Suppression of pro-inflammatory genes (IL-1β, IL-6, TNF-α) in primary human HIV-positive macrophages, as well as feline immunodeficiency virus (FIV)-positive felines	[218]
Inhibits acute LPS-induced microglia-mediated inflammation both in vivo and in vitro through the activation of TrkA-Akt1/2-CREB-Jmjd3 pathway and reduces IL-6, TNF-α, IL-12 and MCP-1 gene expression	[64]

From these studies, it seems that DHEA exerts its anti-inflammatory effects primarily by modulating either the effects or production of pro-inflammatory cytokines. However, it also appears that the same pro-inflammatory cytokines have the ability to regulate DHEA production in turn. For example, in macrophage-depleted primary murine leydig cell cultures, both IL-1 and TNF-α were shown to inhibit androgen steroidogenesis by downregulating the expression of cytochrome P450c17 mRNA—which encodes for an enzyme that is critical to androgen formation [65, 66]. TNF-α has shown similar inhibitory effects in cultured porcine leydig cells by reducing the binding of luteinising hormone (LH) or human chorionic gonadotropin (hCG) to the respective receptor—which would stimulate steroidogenesis [67]. These authors further suggested that the primary inhibitory effect of TNF-α was mainly by decreasing availability of cholesterol, the initial substrate required for steroidogenesis [67].

Regardless of how cytokines may inhibit the synthesis of androgens, these studies provide a plausible explanation for the age-related decline of DHEA not only systemically, but centrally as well—given the relatively pro-inflammatory status associated with ageing. Furthermore, since it has been shown that the inflammatory maladaptation seen with ageing affects the brain to a larger extent (specifically the hippocampus), it is plausible that the age-related decline of DHEA occurs even more rapidly in the brain than it does the rest of the body.

In contrast to the idea that DHEA is largely beneficial, a minority of studies have suggested that DHEA may have neurotoxicity under specific conditions. In a model of global cerebral ischaemic insult in rats [68], intraperitoneal administration of a supraphysiological dose of DHEA 3 to 48 h after ischaemic injury was neuroprotective (e.g. decreased neuronal death in the CA1 hippocampal region). However, DHEA administration 1 h before or after the insult exacerbated the effects of the injury. Given the known anti-inflammatory role of DHEA, this may suggest that (at least at this high dose) DHEA inhibited early inflammatory or oxidative responses that were required for cytoprotection. Additionally, decreased cell viability was reported in primary murine neuronal cultures and human neuroblastoma (SK-N-SH) cells after (24–72 h) exposure to a large dose-range of DHEA—a result not seen in mixed neural or glial cultures. Furthermore, the neurotoxic effects of DHEA were abrogated by simultaneous treatment with DHEAS [69]. From this, it is difficult to formulate an interpretation on potentially toxic effects of DHEA. On the one hand, it is certainly feasible that a high dose of an antioxidant such as DHEA may have damaging effects, as this has been illustrated in the nutraceutical literature [70]. On the other hand, the second study clearly highlights the limitations of interpretations possible from single cell culture models, although it does provide insight on the complexity of the mechanisms at play. In our opinion, given the relatively larger body of evidence in support of a beneficial effect of DHEA, significant concern is probably not warranted in our context of intracrine DHEA production.

In order to further our understanding of how DHEA may impact on the ageing brain, it is necessary to delve deeper into the potential molecular actions and interactions of DHEA and DHEAS.

## Potential mechanisms for DHEA-mediated neuroprotective effects

There are at least two ways via which DHEA can facilitate its effects centrally. Through following the conventional steroidogenesis pathway, DHEA may be converted to endpoint sex hormones, which would then activate their respective receptors. Reviews by different groups have suggested that DHEA can elicit genomic effects through its conversion into testosterone and dihydrotestosterone (DHT)—thereby activating androgen receptors (AR)—as well as through its conversion into estradiol and the subsequent activation of oestrogen receptors (ER) [4, 6]. In support of this suggestion, it has indeed been demonstrated that isolated neonatal rat cortical and hypothalamic astrocytes can synthesise both testosterone and oestrogen from exogenous DHEA [71]. Alternatively, DHEA itself may be able to directly bind to and activate these hormone receptors. Although no cell membrane-bound or nuclear receptor has been described to have an affinity specifically for DHEA or DHEAS, a recent comprehensive review has revealed that DHEA and DHEAS have affinity for a number of membrane and nuclear receptors and binding sites [72], allowing DHEA to directly activate receptors in the brain and CNS specifically. DHEA is known to interact with the cell membrane receptors γ-aminobutyric acid type A (GABA_A_), *N*-methyl-*D*-aspartate (NMDA) and sigma-1 (σ-1), as well as nuclear receptors such as peroxisome proliferator-activated receptor (PPAR)-α and finally neurotropin receptors such as tropomyosin receptor kinase (Trk)-A, Trk-B and Trk-C [64, 72–74]. These potential mechanisms are briefly discussed in the following paragraphs.

Firstly, GABA_A_ receptors are ligand-gated chloride and bicarbonate channels that induce hyperpolarisation to inhibit the postsynaptic potentials when activated [72]. DHEA and DHEAS (to a greater extent) have both been shown to act as non-competitive antagonists of ionotropic GABA_A_ receptor-mediated activity in isolated neurosynaptosomes from Sprague–Dawley rat brains [75]. Similarly, DHEAS was shown to block recombinant GABA_A_ receptor (GABA_A_ R)-mediated currents in transfected HEK293 cells [76, 77]. Another study of reversible spinal cord injury in rabbits showed that DHEAS significantly delayed the onset of ischemia-induced paraplegia [78]. However, despite widely reported, the exact mechanisms of how DHEA or DHEAS may elicit neuroprotective effects through GABA_A_ receptors still remain to be fully elucidated. It is important to point out that DHEA appears to induce multiple effects through GABA receptors—of note and in the context of ischemia injuries it demonstrates antioxidant capacity, which is already accepted in literature and highlights the beneficial pleiotropic effects of DHEA.

Secondly, the NMDA receptor (NMDAR) is a cell membrane bound receptor that is composed of a large family of glutamate receptors. Glutamate receptors bind to neurotransmitters and allosteric effectors which regulate transmembrane ion channels involved in learning and memory, i.e. LTP [72]. DHEAS has been shown to activate NMDARs in both rat hippocampal slices and cultured mouse embryonic cultured neocortical neurons [79, 80]. These reports suggest a more active function of DHEA in the context of NMDARs.

Thirdly, sigma-1 (σ-1) receptors are associated with cellular membranes, endoplasmic reticulum, nuclear membranes and mitochondrial membranes of many cells of neural origin, including astrocytes, oligodendrocytes and microglia [72]. DHEA activation of sigma-1 receptors improved memory deficits in induced in mouse models [81]. In addition, the effect that DHEA and DHEAS have on NMDARs appears to be linked to sigma-1 receptor interactions. Evidence has shown that DHEAS can potentiate NMDA evoked release of norepinephrine in rat hippocampal brain slices through its action as a sigma-1 agonist [82].

Fourthly, peroxisome proliferator-activated receptors or PPARs are nuclear-associated transcription factors that are capable of exerting pleiotropic physiological effects, such as regulating lipid metabolism, participating in glucose homeostasis and even having a role in apoptosis [83]. DHEA and DHEAS may exert neuroprotective effects at nuclear receptors such as PPARα. Specifically, inhibiting NF-kB binding to PPARα. In a rodent study, the effects of DHEA and DHEAS treatment in aged wild type (PPARα^+/+^) vs aged PPARα knockout mice (PPARα^−/−^) were investigated. The aged wild-type mice treated with DHEA and DHEAS exhibited reduced tissue lipid peroxidation, reduced NF-kB activation in the spleen and lower pro-inflammatory cytokine production when compared to knockout mice also treated with DHEA and DHEAS [84]. It is worth noting though that no specific binding affinity of PPAR for DHEA or DHEAS has been reported [85]. Despite this fact, PPARα receptors have increasingly been reported to have a role in mediating oxidative stress and inflammation in brain pathologies such as traumatic brain injury (TBI) [83, 86].

In line with these mechanisms, DHEA has been shown to activate the pro-survival phosphatidylinositol-4,5-bisphosphate 3-kinase/protein kinase B (PI3/Akt) pathway. In fact, a study investigating the effects of NMDA-induced excitotoxicity in culture mouse brain cells, illustrated that DHEA was able to mitigate the detrimental effects by activating the PI3/Akt pathway through a calcium dependent mechanism [87]. Similarly, DHEA supplementation in aged Wistar rats was able to increase the expression of phosphorylated-Akt in liver tissue [88].

In terms of neurotropin receptors, DHEA has been shown to bind with high affinity to the neurotrophin growth factor (NGF) receptor TrkA in HEK293 cells transfected with TrkA plasmid cDNA [73]. Binding of DHEA to TrkA led to phosphorylation of the receptor and activation of downstream pathways such as the Akt, extracellular signal-regulated kinases (ERK)-1/2 and SHC-transforming protein (Shc) signalling cascades [73]. In a similar study, also conducted in transgenic HEK293 cells, DHEA was shown to also bind to TrkB, which is mainly activated by brain-derived neurotrophic factor (BDNF), and TrkC, which is preferentially activated by neurotrophin-3 (NT-3) [74]. However, this study showed that DHEA binding only led to the phosphorylation and activation of TrkC, but not TrkB [74]. It was noted that DHEA bound these receptors by two orders magnitude lower than that of the inherent neurotropins.

Finally, DHEA may also have a modulatory role in cytoskeletal dynamics. In this context, DHEA has been found to bind to the N-terminal of the dendritic localised microtubule associated protein type 2C (MAP2C). MAPs bind to and facilitate the dynamic microtubulin polymerisation and DHEA was reported to increase the length of dendrites expressing MAP2C [89]. This finding is of particular relevance in the context of neurodegenerative pathologies such as AD where a pathological change in neuritic morphology is associated with disease [90].

Taken together, these studies indicate a significant modulatory role of DHEA/DHEAS via its binding to a wide variety of receptors. Of particular relevance to the current topic, DHEA-linked anti-inflammatory—albeit mainly demonstrated in circulation—and neuroprotective outcomes indirectly suggest that several benefits may come from prevention of DHEA decline. Supplementation is one clear avenue through which this may be achieved.

### Efficacy of therapeutic DHEA administration

DHEA supplementation has historically been used in the treatment of reproductive-related diseases, particularly in women, where it has been shown to alleviate menopause-related pathologies such as vaginal atrophy [91]. In support of our interpretation of benefit from maintaining DHEA availability, in diseases not related to reproduction, DHEA supplementation has been shown to be beneficial to both sexes in the treatment of numerous other pathologies or conditions (see Table 4).

**Table 4:** Illustrating the variety of studies reporting favourable effects of DHEA/DHEAS supplementation in clinical pathologies

Disease state	References
Autoimmune disease	
Systemic lupus erythematosus	[219–227]
Metabolic disease	
Hypercholesterolemia	[228]
Metabolic syndrome	[229]
Genetic diseases	
Hereditary angioedema	[230]
Ageing-associated	
Advanced age	[231, 232]
Osteoporosis	[233, 234]
Endocrine disorders	
Hypopituitarism	[235–238]
Adrenal insufficiency/Addison’s disease	[239–246]

Despite these many clinical studies involving DHEA supplementation, to the best of the authors’ knowledge, the effects of exogenous DHEA supplementation has not been directly assessed in clinical manifestations of neurodegeneration or any related pathologies. Similarly, there are also currently no clinical studies assessing the effects of DHEA or DHEAS supplementation as an adjuvant therapy for neurodegenerative diseases in either the European or American clinical trial database.

In addition, it should be noted that other studies have reported oral DHEA supplementation to show little to no benefit in the treatment of other ageing-related pathologies such as cognitive performance, lipid metabolism, glucose metabolism, bone health and muscle function [92–94].

However, the available clinical studies collectively do provide some insight. The conditions included in our summary (Table 4), which all suggests benefit from DHEA administration, are linked by the fact that inflammation is a characteristic feature in all. Similarly, neuroinflammation is a critical hallmark of the generalised pathophysiology of neurodegeneration and its occurrence appears to have a causative role in this degenerative condition. Thus, given the already discussed anti-inflammatory effect of DHEA (refer to Table 3), as well as the anti-inflammatory therapeutic effect suggested by the results just presented, theoretically, if one can increase DHEA production at cellular level in the central compartment, one should be able to alleviate or prevent neuroinflammation and thus neurodegeneration.

### Is it possible to stimulate extragonadal synthesis of DHEA or DHEAS?

Since exogenous DHEA supplementation has yielded varying benefit, an obvious alternative therapeutic—or preventative—strategy to consider is whether it might be naturally or artificially possible to increase endogenous DHEA production.

From the sport and exercise literature, it is known that exercise in older males can significantly increase serum DHEA and DHEAS concentrations [95–101]. These results do not provide proof of extragonadal androgen synthesis though, as moderate exercise is known to increase gonadal testosterone production. However, interestingly, similar effects of exercise have been reported for DHEA in older, post-menopausal females [102–104]. In this population, where gonadal androgen production is less possible, it is possible that extragonadal DHEA production may occur in response to exercise.

Interestingly, a study conducted in Sprague-Dawley rats has illustrated skeletal muscle to have endogenous steroidogenic capacity and that acute bouts aerobic exercise can significantly enhance the localised production of DHEA in both male and female animals [105]. This adds substantial support for our theory that extragonadal DHEA synthesis may be elicited through intervention.

Furthermore, higher DHEAS levels have been reported in long-term practitioners of alternative therapies such as transcendental meditation and the ancient martial art of tai chi, when compared to age-matched controls [106, 107]. Although these studies do not prove that DHEA production is stimulated by these practices, the relatively higher levels suggest at least an effect to sustain long-term production rate. In line with these results obtained in psychology-based practices, environmental enrichment in caged rats was linked to increases in steroidogenic enzyme expression associated with DHEA production, relative to unstimulated rats of the same age [108]. Indeed, enrichment therapy is already being employed to treat patients with dementia and Alzheimer’s disease [109–113], although the authors could not find data on the effect of this strategy on DHEA synthesis.

Considering this evidence, perhaps it is necessary to first understand which tissues may possess the ability for DHEA synthesis, before considering the feasibility of this approach in the context of neurodegeneration.

### DHEA biosynthesis

DHEA is produced during steroidogenesis, in a manner dependent on mainly two classes of steroidogenic enzymes, namely the P450 enzymes and the hydroxysteroid dehydrogenases [114]. Also, DHEA can be sulphated into DHEAS via enzymes called steroid sulphotransferases (SULT), while conversion of DHEAS back into DHEA is mediated via steroid sulphatases (STS). DHEAS levels continue to increase after puberty, peaking in the mid-20s, after which levels begin to progressively decline with age in both men and women [3, 12]. Serum DHEA and DHEAS levels are thought to be almost exclusively maintained by their synthesis in the adrenal zona reticularis [114]. However, in theory any tissue that expresses the steroid enzymes can be classified as steroidogenic. In fact, in the early 80s Fernand Labrie, a well-known endocrinologist, was the first to note that prostate glands of patients with prostate cancer still had high levels of dihydrotestosterone (DHT) even after castration [115]. He illustrated that some peripheral tissues, such as the prostate, possesses the steroidogenic enzymes needed to transform DHEA and DHEAS into its androgenic and estrogenic metabolites, thereby capable of exerting a relatively more localised effect. The study of this unique ability of peripheral tissues was termed “intracrinology” and has subsequently gained some momentum [116]. Given the importance of intracrine derived hormone production in terms of female reproductive health, it is clear that investigations into intracrinological mechanisms in other contexts are warranted, such as in neurodegeneration and ageing.

Although it is known that intracrinology takes place in peripheral tissues, all tissue types do not express the same isoforms of the steroidogenic enzymes, nor are they expressed in the same quantity or constitutively expressed as we age. For example, a recent study in rats demonstrated that the expression of several steroidogenic enzymes decline significantly in the brain with age [108]. It is important to have an understanding of where the capacity for steroidogenesis exist, as well as to consider sex and species differences, as these aspects significantly impact on choices for experimental models, as well as on the relevance and applicability of research data gained in any particular model. It is not possible to address steroidogenesis in its totality within the scope of this review. Rather, in the next few sections we describe some of these intricacies in the context of DHEA synthesis specifically.

### Production sites of DHEA

In terms of substrate delivery for steroidogenesis, the steroidogenic acute regulatory protein (StAR) and cholesterol side-chain cleavage enzyme (P450scc) are required for the transport of cholesterol to the mitochondria and conversion into pregnenolone, respectively. Interestingly, these enzymes are expressed at significantly lower levels in the Leydig cells of aged rat testes relative to those of younger rats [117], which—since Leydig cells are the primary cells for testosterone production in the male body—provides a plausible explanation for the age-related decline of the hormone in both rodents and humans. Interestingly, in contrast, P450scc protein and mRNA expression in the human adrenal glands appears to be relatively stable with ageing [118, 119].

**Fig. 1:**
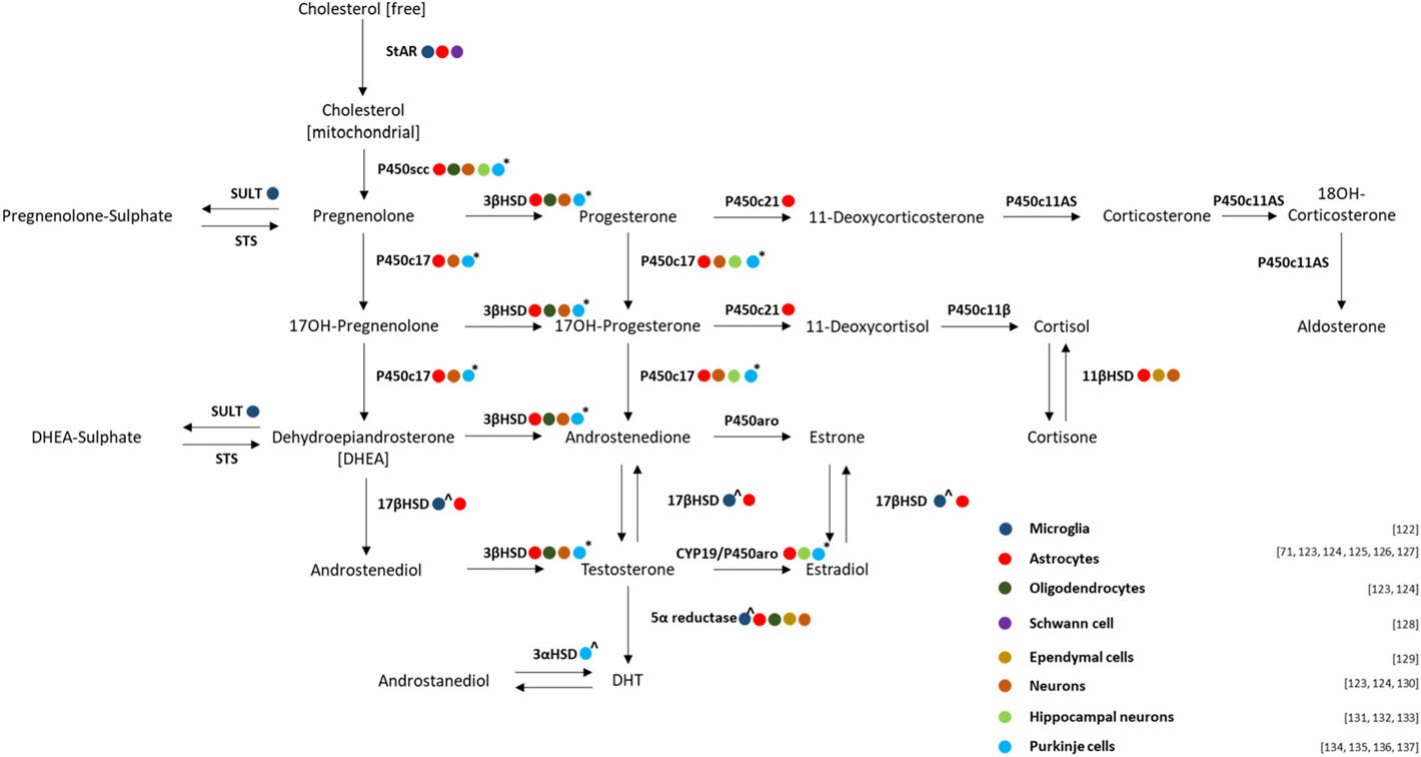
Representative diagram of reported steroidogenic enzyme expression reported in different brain cell types. Species employed were rat, unless otherwise additionally indicated (^mouse or *canine) [71, 122–137]

In the context of central intracrine production of DHEA, P450scc mRNA has been detected in cultured human astrocytes and oligodendrocytes (but not neurons) in at least one study [120]. Many other studies have detected steroidogenic enzyme expression in both neurons and neuroglial cells in vertebrates other than humans (refer to Fig. 1). We tabulated these studies for brevity, but it is important to note that although steroidogenic enzyme mRNA was detectable in these different animal brain cell types, it is not clearly indicated to what extent the level of gene expression may differ from one cell type to another. Our interpretation of the data presented in most of these cited studies, is that neuroglial cells appear to express higher levels of steroidogenic enzyme mRNA relative to neurons, where levels were barely detectable in some cases. This suggests that mainly neuroglial cells are capable of significant intracrine synthesis, as already reported for peripheral tissue other than gonads and adrenals [57, 58]. In contrast to the P450scc findings, StAR mRNA expression in rat brain brains has been shown to indeed decline significantly with age [121], suggesting a relative decline in substrate delivery similar to that reported for the testis. Whether this occurs in human brain tissue could not conclusively be answered from the literature, but it certainly cannot rule out. Nevertheless, the presence of both StAR and P450scc in astrocytes and oligodendrocytes argues in favour of sufficient substrate availability to indeed allow for localised production of DHEA in these cells.

Another protein of potential relevance in the context of cholesterol transport to the mitochondria has been described. Translocator protein (TSPO), also known as peripheral-type benzodiazepine receptor (PBR), a protein mainly bound to the outer mitochondrial membrane, has also been suggested to play a role in the facilitation of cholesterol transport via StAR [138]. Whether the presence of TSPO is critical in the transport of cholesterol is a matter of controversy within literature. It was shown in mice, with a specific TSPO gene knockout in their Leydig cells, that testosterone could still be produced despite the absence of what thought to be a critical protein for the process [139]. Despite the uncertainty surrounding its role in steroidogenesis, TSPO has recently been identified as a potential biomarker of neurodegeneration, as its expression increases with inflammation and neurodegeneration associated with Alzheimer’s disease, HIV encephalitis and MS [140, 141].

More specifically in terms of its function centrally, in vitro experiments in primary human astrocytes have demonstrated that various synthetic TSPO ligands of the *N*,*N*-dialkyl-2-phenylindol-3-ylglyoxylamide class (PIGAs) are capable of activating TSPO activity and stimulating neurosteroidogenesis [142]. In fact, PIGAs have shown to be neuroprotective in l-buthionine-(S,R)-sulfoximine (BOS) induced cytotoxicity of C6 rat glioma cells [143]. The mechanism of neuroprotection was associated with a reduction in lipid peroxidation and inflammation, which was abrogated in presence of the P450scc and StAR inhibitor aminoglutethimide and was therefore thought to occur due to steroidogenesis. Given the known anti-inflammatory and antioxidant properties of DHEA, TSPO may be a role player in the maintenance of DHEA levels.

Another fact in favour of local DHEA production in the brain is the relatively increased demand for DHEA in brain relative to the periphery. Firstly, steroid sulphotransferase isoform 2A1 (SULT2A1)—which increases circulating DHEAS by sulphating DHEA, rendering it bio-inactive [118]—seems to not be present in brain tissue at all, while SULT2B1 expression has only been reported for the prefrontal cortex, hippocampus and cerebellum, but not for the cerebral subcortex or neocortex [144, 145]. Secondly, sulphatase (STS) expression—which is responsible for conversion of DHEAS into the bioactive DHEA—is generally very high in the CNS [6]. Together, this suggests a relatively higher demand for DHEA in the brain when compared to the periphery. This scenario would also increase the likelihood of mechanisms for local production to be in place.

The idea of de novo synthesis of DHEA and DHEAS in the brain itself remains a highly debated topic in the field. As mentioned, the human adrenal glands were initially thought to be the only site of endogenous steroidogenesis, apart from the gonads (i.e. testes, ovaries) which produce DHEA and DHEAS to a lesser extent. However, the brain has recently and controversially been highlighted as a possible alternative site for production. Given the neuroprotective and central anti-inflammatory effects of DHEA already discussed, this possibility for intracrine DHEA production has far-reaching implications for both neurodegenerative disease and ageing—not only to increase our understanding of the disease processes, but also in terms of development of preventative and/or therapeutic strategies.

The first suggestion of DHEA synthesis in the brain was already published in the 1980s—when DHEA and DHEAS concentrations were reported to be higher in rat brains than in circulation [146]. A few years later, similar circumstantial evidence for DHEA synthesis in the human brain emerged [147]. DHEA was subsequently commonly referred to as a “neurosteroid”, based on its relatively increased central localisation. Although DHEA can be detected in both animal and human brain tissue, it remains unclear whether de novo synthesis indeed occurs. For evident reasons, it is practically difficult to accurately assess actual DHEA production in the human brain, so that most data is generated from rodent models. To add complexity to this topic, subsequent evidence may suggest that a species difference in terms of central DHEA levels may exist. In terms of criticism of analytical techniques, it has for example been suggested that DHEA levels in rat brain tissue reported in earlier studies may have been overestimated due a lack of sensitivity of the radio-immunoassays (RIA) technique used at the time, which relied on antibody specificity to detect the steroid hormones [148]. A newer method encompassing solid phase extraction (SPE), high-performance liquid chromatography (HPLC) and gas chromatography-mass spectrometry (GC-MS) was developed to generate more accurate data [148]. Ironically, nearly a decade later it was reported by the same authors that this technique may indeed also be flawed when it came to the detection of sulphated steroids [149]. They showed in rodent samples that the levels of DHEA and PREG were higher in the lipoidal fraction of the SPE. It was suggested that cholesterol contamination may have contributed to these findings through cholesterol autoxidation, generating DHEA and PREG, and that actual levels of DHEA and DHEAS are likely extremely low or essentially non-existent in rat brain tissue [149]. Interestingly, in the same paper, the authors were able to confirm the presence of significant amounts of both DHEA and PREG in human brain tissue samples using this improved methodology. The exact mechanisms of how cholesterol autoxidation leads to DHEA and PREG generation remains to be elucidated. Nevertheless, these results highlight the necessity of considering species in the design of experimental models. The fact that DHEA cannot be detected in rodent brains may be a function of generally lower levels when compared to humans. Thus, although rodents can still be feasible models, the parameters to be assessed, might have to differ depending on analytical sensitivity.

Given these methodological limitations in the measurement of hormone levels directly, many indirect methods have been employed to investigate the possibility of DHEA production centrally. However, results have again posed more questions than answers. For example, on one hand, P450c17 mRNA—which transcribes an enzyme that is crucial in the formation of DHEA—has been detected in cultured human glial cells [120]. Subsequently, P450c17 mRNA expression has also been demonstrated in the amygdala, caudate nucleus, cerebellum, corpus callosum, hippocampus and thalamus of the human brain [119]. In addition and similarly to circulating DHEA levels, the enzyme expression also declines with age in the rat brain, which argue in favour of the possibility for local DHEA synthesis [150]. Interestingly, the age-related decline in P450c17 mRNA was reported to be more pronounced in the cerebral cortex and cerebellum, but not the hypothalamus [150]—this fact should be further explored in order to determine its relevance.

However, the expression of P450c17 in the human brain has been a topic of debate – at least two other studies could not detect any P450c17 mRNA in the human cerebellum, hippocampus or temporal lobe and reported no P450c17 activity in the temporal lobe [145, 151]. There are a variety of possible explanations for these different results. Firstly, it is known that age has a significant effect on steroidogenic enzyme expression, so it is possible that this may have affected the results. Indeed, in the study where the P450c17 mRNA was detected, samples were pooled from patients between the ages of 10 to 78 years. On the other hand, in at least one of the studies failing to detect enzyme activity, samples were collected from a much older patient cohort (80 years and older) [119, 151]. Secondly, in terms of the failure of any group to report activity of the enzymes which catalyse the reaction in the brain [6], there is currently no way to determine at which rate potential local DHEA synthesis would occur. An important consideration here is that P450c17 expression reported for the CNS is substantially lower than for the testes, which exceeds it by nearly 200-fold [6]. It is therefore likely that the conventional methods previously employed in studies with negative outcome, may not have been sufficiently sensitive to detect these possibly very low levels of activity. Thus, the issue remains to be elucidated across the ageing spectrum, to eliminate the potential for a false negative outcome by using aged individuals.

Additionally, it is possible that localised DHEA biosynthetic mechanism in the brain may differ from that at other sites of local DHEA production. One group has indeed suggested that DHEA and DHEAS can be synthesised in manner independent of P450c17. Their evidence suggests that cultured human astrocytes and oligodendrocytes, but not neurons, could synthesise DHEA the presence of a P450c17 and 3β-HSD inhibitor. It is suggested that this alternative pathway may be dependent on reactive oxygen species and produce DHEA from an unknown “precursor” molecule other than pregnenolone [120]. Although this study provided interesting data, there is a limitation to the scientific rationale, as the authors admitted that the P450c17 inhibitor used could not block endogenous DHEA production in the glial cells. At least one other lab has also speculated on the possibility for an alternative pathway of DHEA production linked to free radicals [152]. This is not impossible, since a precedent in this regard exists: an alternative steroid production mechanisms in the brain was clearly demonstrated in the case of 21-hydroxylation, which appears to be mediated through CYP2D instead of CYPC21 [153].

Apart from the debate on the capacity for intracrine DHEA synthesis in the CNS, several theories have been formulated in attempts to explain the relatively higher DHEA and DHEAS concentrations in the central compartment. For example, it has been suggested that DHEA in the brain may be produced from DHEAS delivered from circulation, through conversion by steroid sulphatase (STS). Indeed, it has been shown that the human temporal lobe expresses high amounts of STS mRNA as well as activity in the cerebral cortex relative to subcortical white matter [145]. However, there are two arguments against this hypothesis. Firstly, sulphated hydroxysteroids such as DHEAS are hydrophilic and do not readily cross the blood–brain barrier [154]. Although sulphated steroids such as DHEAS and pregnenolone sulphate may enter the brain through circulation via organic anion transporting peptides (OATP) transporting them both ways, it would appear that OATP transport of sulphated steroids favours movement out of the CNS, not in [155]. However, a recent study investigating the transport of DHEAS and PREGS through the blood brain barrier (BBB) of rats found that although PREGS entered the blood brain barrier more readily, DHEA was rapidly metabolised into androstenedione and androstenediol [156]. Further investigation found that the enzymes responsible for the rapid desulphatasion were mainly localised the BBB capillaries as opposed the brain parenchyma [156]. This argues that the DHEA in brain is likely from peripheral sources as opposed to de novo synthesis. However, it is important to note that the overall efflux of DHEAS through the BBB may still not be rapid enough to account for the differences in concentration in the brain versus the periphery.

Secondly, as already mentioned, DHEA and DHEAS concentrations have been reported to be markedly higher in the human brain than in circulation [147]. In this study, DHEA concentrations were determined in the brains of 10 subjects (9 females and 1 male, ages ranging from 80 to 93). Nevertheless, in rat brains DHEA concentrations were higher relative to circulation and remained so even 15 days after an adrenalectomy and/or orchiectomy [146]. This argues against the idea of systemic delivery. However, as mentioned earlier the techniques that were used to determine the concentration of DHEA in these studies may reassessed due to possible cholesterol contamination.

Taken together there still exists a strong body of evidence to support that de novo occurs in the brain. Although it is near impossible to prove this specifically in humans, as one will be faced with clear practical and ethical hindrances, it is the opinion of the authors that the human brain does indeed produce DHEA centrally. In order to understand how DHEA may be produced in the brain, the use of animal models may be the only way to elucidate key pathways. This, however, does not come without its challenges.

### Practical challenges in steroidogenesis research

Several practical challenges have already been alluded to in this review, such as methodological issues pertaining to mRNA and protein determination, as well as gender-specificity. A remaining consideration which will be touched on in this section, is the importance of species selection for investigations of this nature.

**Fig. 2:**
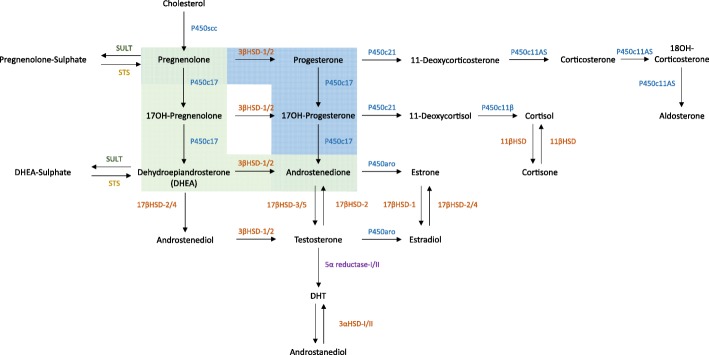
The process of steroidogenesis. Cytochrome P450 enzymes (blue font), hydroxysteroid dehydrogenases (orange font), reductases (purple font), and steroid sulphatases (yellow font) and sulphotransferases (green font). Part of the pathway highlighted in green denotes the Δ5 pathway, which is favoured in human steroidogenesis, and the blue denotes the Δ4, which is favoured in rodent steroidogenesis

For example, it is important to consider that systemic steroidogenesis in humans, higher primates and bovines differ from rodents in two significant ways. Firstly, humans primarily convert PREG to androstenedione through the Δ^5^ pathway in the following manner: PREG » 17OH-PREG » DHEA » androstenedione. Rodent steroidogenesis on the other hand favours the Δ^4^ pathway; PREG » progesterone » 17OH-progesterone (17OH-PROG) » androstenedione (Fig. 2). This is speculated to be the reason why humans have high levels of circulating DHEAS, while in rodents levels are significantly lower when compared to humans [157].

Secondly, in humans the adrenal gland is the main source of DHEA production, but in rodents the gonads are the main source, as the adrenal glands of most species mice and rats appear to lack P450c17 enzyme activity [158, 159]. Although human gonads do produce androgens such as DHEA, the reason for adrenal preference of androgen production is not clear. The use of rodent models in studying the systemic role DHEA has thus garnered concern due the significant differences between humans and rodents. However, the fact that DHEAS has been found in both human and rodent brain tissue suggests that potential for de novo DHEA production exists in the brain in both species. Therefore, rodents could still serve as reliable model systems in studying DHEA and its role in the brain.

Of specific interest in this context, it was recently discovered that the precocial species of mouse, *Acomys cahirinus* or Spiny mouse, possess adrenal glands that express P450c17 and are in fact capable of producing DHEA [160, 161]. This unique mouse has a longer gestational period (± 39 days) when compared to other mice and rats and unlike other rodents, has a brain growth pattern that is comparable with humans at the time of birth [162, 163]. It has also been shown in spiny mice that cortisol is the main glucocorticoid in the blood, unlike in rats where corticosterone is the main circulating glucocorticoid [160, 161]. Most importantly, explanted foetal, neonate and adult spiny mouse brain tissue in culture have been shown capable of producing DHEA in the presence of pregnenolone [164]. In addition, spiny mouse also exhibit an age-related decline in DHEA synthesis [163]. These mice may prove to be a particularly reliable and relevant model for studying the mechanisms of DHEA in many neurodegenerative disease states.

## Conclusions

Based on the successes reported in experimental model systems, DHEA has been proven to be beneficial to limit progression of various inflammation-associated neurodegenerative diseases. From our review of the relevant literature, we conclude that *central*, intracrine synthesis of DHEA is highly probable and we have highlighted several mechanisms by which locally synthesised DHEA may affect preventative and/or therapeutic benefit in the context of neurodegeneration. Current literature is limited by controversies regarding methodological accuracy and the suitability of basic neuronal cell culture models. Taken together, this stresses the urgency for in vivo research specifically focused at not only substantiating results from experimental models, but also at elucidating in detail how these processes may be harnessed for therapeutic benefits.

## Abbreviations

17OH-PREG: 17-Hydroxy-pregnenonolone
3-NP: 3-Nitropropionic acid
AD: Alzheimer’s disease
AR: Androgen receptor
BBB: Blood–brain barrier
BDNF: Brain-derived neurotrophic factor
CD11b: Complement receptor 3
CNS: Central nervous system
CS: Corpus striatum
CSF: Cerebral spinal fluid
CYP2D: Cytochrome P450 2D
DHEA: Dehydroepiandrosterone
DHEAS: Dehydroepiandrosterone sulphate
DHT: Dihydrotestosterone
*E. coli*: *Escherichia coli*
EAE: Experimental autoimmune encephalomyelitis
ER: Oestrogen receptor
ERK 1/2: Extracellular signal-regulated kinases-1/2
FIV: Feline immunodeficiency virus
GABA_A_ R: GABA_A_ receptor
GABA_A_: γ-Aminobutyric acid type A
GC-MS: Gas chromatography mass spectrometry
GR: Glucocorticoid receptor
hCG: human chorionic gonadotropin
HPA: Hypothalamic pituitary adrenal axis
HPLC: High-performance liquid chromatography
HSD: Hydroxysteroid dehydrogenase
IFN-γ: Interferon gamma
IL: Interleukin
LH: Luteinising hormone
LPS: Lipopolysaccharide
LTP: Long-term potentiation
MAP2C: Microtubule-associated protein type 2C
MCP-1: Monocyte chemoattractant protein 1
MHC II: Major histocompatibility complex II
MOA: Monoamine oxidase
MR: Mineralocorticoid receptor
mRNA: Messenger ribonucleic acid
MS: Multiple sclerosis
NAc: Nucleus accumbens
NF-κB: Nuclear factor kappa-light-chain-enhancer of activated B cells
NGF: Neurotrophin growth factor
NMDA: *N-*methyl*-D-*aspartate
NMDAR: NMDA receptor
NT-3: Neurotrophin 3
OATP: Organic anion transporting peptide
P450: Cytochrome P450 enzyme
P450scc: Cytochrome P450 side chain cleavage enzyme
PBMC: Peripheral blood mononuclear cell
PD: Parkinson’s disease
PI3/Akt: Phosphatidylinositol-4,5-bisphosphate 3-kinase/protein kinase B
PPAR: Peroxisome proliferator-activated receptor
PREG: Pregnenolone
PREGS: Pregnenolone sulphate
PTSD: Post-traumatic stress disorder
RIA: Radio-immunoassay
SAM: Sympatho-adrenal-medullary
Shc: SHC-transforming protein
SNS: Sympathetic nervous system
SPE: Solid phase extraction
StAR: Steroidogenic acute regulatory protein
STS: Steroid sulphatases
SULT: Steroid sulphotransferase
TNF: Tumour necrosis factor
Trk: Tropomyosin receptor kinase
σ-1: Sigma 1
